# Depuration of Polybrominated Diphenyl Ethers (PBDEs) and Polychlorinated Biphenyls (PCBs) in Breast Milk from California First-Time Mothers (Primiparae)

**DOI:** 10.1289/ehp.10166

**Published:** 2007-06-28

**Authors:** Kim Hooper, Jianwen She, Margaret Sharp, Joan Chow, Nicholas Jewell, Rosanne Gephart, Arthur Holden

**Affiliations:** 1 Environmental Chemistry Laboratory, California Department of Toxic Substances Control, Berkeley, California, USA; 2 Division of Biostatistics, School of Public Health, University of California, Berkeley, California, USA; 3 Woman’s Health and Birth Center, Santa Rosa, California, USA

**Keywords:** breast-feeding, breast milk, brominated flame retardants, depuration, exposure assessment, lactation, PBDEs, PCBs

## Abstract

**Background:**

Little is known about the rates of loss (depuration) of polybrominated diphenyl ethers (PBDEs) and polychlorinated biphenyls (PCBs) from mothers during lactation. Depuration rates affect infant exposure to chemicals during breast-feeding, and fetal and lactational transfers during subsequent pregnancies.

**Objective:**

Our objective in this study was to estimate depuration rates of PBDEs and PCBs using serial samples of breast milk.

**Method:**

Nine first-time mothers (primiparae) each collected samples at 4, 6, 8, 12, 16, 20, and 24 weeks after birth. Nine additional primiparae each collected two samples at varying time intervals (18 to > 85 weeks after birth). Analytical precision was assessed to evaluate the accuracy of measured monthly percentage declines in PBDEs and PCBs.

**Results:**

The four major PBDE congeners decreased 2 or 3% ± 1% per month over the 6-month period. These decreases were consistent over a 50-fold range (21–1,330 ng/g lipid weight) of initial PBDE concentrations in breast milk. The change in PCB-153 ranged from + 0.3% to –0.6% per month, with heterogeneous slopes and greater intraindividual variability. PBDE and PCB concentrations declined 1% per month over longer periods (up to 136 weeks).

**Conclusions:**

Our data indicate that PBDEs and PCBs are not substantially (4–18%) reduced in primiparae after 6 months of breast-feeding. Consequently, the fetal and lactational exposures for a second child may not be markedly lower than those for the first. Participants were volunteers from a larger study population (*n* = 82), and were typical in their PBDE/PCB levels and in many demographic and lifestyle factors. These similarities suggest that our results may have broader applicability.

Polybrominated diphenyl ethers (PBDEs) and polychlorinated biphenyls (PCBs) are persistent, lipophilic chemicals with different sources, pathways, and patterns of human exposure. Mixtures of PBDEs are present as noncovalently bound flame-retardant additives to synthetic fabrics, foams, or plastics in a variety of consumer products. Two mixtures, the penta- and octa-BDEs, are no longer used. Deca-BDE, the major PBDE with total production approaching that of PCBs at their peak, remains in use but is banned in Sweden, Maine, and Washington State. PBDE levels, where examined, have been increasing in humans and wildlife worldwide, doubling every 2–5 years (Hites 2004; Noren and Meironyte 2000; Sjödin et al. 2003a). PCBs have been banned for 30 years, but are present as dielectrics in telephone pole–mounted transformers common to urban areas. Exposure pathways for PCBs, as with most persistent organic pollutants, begin in the outdoor environment. Exposure pathways for PBDEs begin with indoor environments (Jones-Otazo et al. 2005; Schecter et al. 2005; Sjödin et al. 2003b; Stapleton et al. 2005; Thuresson et al. 2006; Wilford et al. 2005). Little is known regarding the rates of loss (depuration) of PBDEs and PCBs from mothers during lactation. It is of interest to examine depuration of PBDEs and PCBs in breast-feeding mothers to determine whether depuration patterns reflect differences in sources, pathways, and patterns of human exposures. In a review of published data on depuration of persistent chemicals via breast milk, LaKind et al. (2001) indicated that most studies were not specifically designed to measure depuration rates, and were limited by combinations of small sample size (*n* = 1–3), few intra-individual measurements, interindividual measurements, or pooled samples. Preliminary data from an ongoing study suggest low depuration rates for PBDEs and PCBs (Sjödin et al. 2005). Our studies, initiated in March 2003, were designed to assess depuration patterns for PBDEs and PCBs using serial samples of breast milk from lactating mothers.

## Materials and Methods

### Serial and precision studies

We examined depuration rates of 12 PBDE congeners and 80 PCB congeners by measuring concentrations in serial samples collected over extended periods from two groups (*n* = 9 for each)—short-term (ST) and long-term (LT)—each composed of volunteers from a **c**ross-**s**ectional study of California primiparae (CS CA) (*n* = 82) (Table 1). ST participants collected hand-expressed samples at fixed intervals (4, 6, 8, 12, 16, 20, and 24 weeks after infant birth) and a pumped sample at 6 weeks. Some ST mothers missed collections, and one ST mother collected 3 additional monthly samples, for a total of 10 samples over 38 weeks. LT participants collected two samples separated by varying intervals, with initial samples collected 1–6 weeks after birth and final samples 18 to > 85 weeks later.

Breast milk samples were run in batches of 12: nine experimental samples and one duplicate, one method blank, and one quality-control sample from a breast milk pool (QCP). All samples from a participant were run in one batch.

To measure biologically based changes in PBDE or PCB levels in serial milk samples, good analytical precision is required to distinguish anticipated small changes from variations in laboratory background. To assess our precision, we made repeated measurements on identical samples. For ST participants, whose five to seven samples were analyzed as one batch, we analyzed eight identical QCP samples as one batch and calculated the relative SD (RSD; SD over the averaged eight observations, expressed as a percentage). For LT participants, whose two samples were analyzed in one batch, we analyzed duplicate samples (21 for PBDEs, 16 for PCBs) and calculated the RSDs (Table 2). For comparison, we also analyzed 16 pairs of concurrently collected hand-expressed and pumped samples (HE/P) (Table 2).

Participants were healthy, first-time (prim-iparous) breast-feeding mothers with healthy, singleton, 1- to 8-week-old infants and abundant milk supplies (Table 1). We followed institutional review board guidelines, and participants signed approved consent forms.

Mothers collected milk samples (~100 mL each) into chemically clean, 120-mL foil-wrapped or amber glass jars over a 2- to 3-day period. Jars were refrigerated between collections, frozen at completion, transported to the laboratory, and stored at −20°C. Mothers followed written protocols to minimize contamination by skin, hair, or dust. Samples were collected between March 2003 and October 2005.

### Sample analysis

Samples were prepared in four steps (lyophilization, extraction and fat determination, and mixed silica gel column cleanup, and gel permeation chromatography column cleanup), and were analyzed by high-resolution gas chromatography–mass spectrometry. Methods are described elsewhere (She et al. 2007).

We examined patterns of mean PBDE and PCB levels over time using mixed-effects linear regression models (xtmixed program in Stata 9.0; Stata Corporation, College Station, TX) for log values (levels were log-normally distributed). Mixed-effects models allowed for random intercepts and random slopes for the regression of a (log) level against time since birth (Rabe-Hesketh and Skrondal 2005). This allows a unique linear regression relationship between log PBDE measurements and the time since birth for each mother, while assuming that intercepts and slopes for the sampled mothers arise from a population distribution of intercepts and slopes. The variances of this latter distribution indicate quantitatively how much mothers differ in either their intercept or slope, or both. The intercept is the log exposure value at time zero (i.e., birth).

## Results

### Demographics of study populations

Mothers and infants in the ST, LT, and CS CA studies were similar in several demographic, physical, and lifestyle indices (age, race, education, body mass index, smoking status, years residency in California, and breast-fed as child, with infants similar in sex ratio as well as birth length and weight) and dissimilar in family income (Table 1).

### PBDE and PCB levels

**Figure 1:**
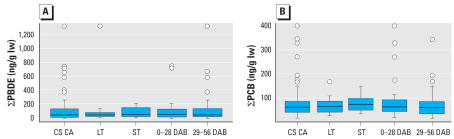
Distributions of ∑PBDEs (*A*) and ∑PCBs (*B*) in breast milk samples from the five study populations of California primiparae: CS CA (*n* = 82); ST (*n* = 9); LT (*n* = 9); 0–28 DAB (*n* = 35); and 29–56 DAB (*n* = 35). lw, lipid weight. ∑PBDE and ∑PCB values for participants in the ST and LT studies are for their initial samples. Lines inside boxes are medians; lower and upper boundaries of boxes are 1st and 3rd quartiles, respectively; and whiskers indicate most extreme data points no more than 1.5 times the interquartile range from boxes. Data points indicate outliers.

∑PBDEs were calculated by summing values for BDEs 32, 28/33, 47, 66, 71, 85, 99, 100, 153, 154, and 183. ∑PCBs summed values for PCBs 110, 114, 118, 105/127, 132/153, 138, 156, 157, 180, and 170/190. ∑PBDE and ∑PCB levels and distributions in the ST, LT, and CS CA populations were similar (Figure 1). BDE-47 and PCB-153 were the major congeners in all three studies and correlated with ∑PBDEs (*r* = 0.99) and ∑PCBs (*r* = 0.89), respectively. Consequently, we have used BDE-47 and PCB-153 values to illustrate depuration profiles.

### Analytical precision

**Figure 2:**
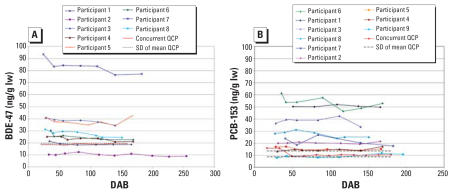
Decreases in BDE-47 (*A*) and PCB-153 (*B*) levels in breast milk collected by ST participants 6–24 weeks after birth. lw, lipid weight. For BDE-47, only 8 ST participants are shown; the ninth participant had a much higher (542 ng/g lw) initial BDE-47 concentration, but her depuration slope was similar. To illustrate measurement precision, the 8 QCP samples that were analyzed concurrently are plotted, with dotted lines showing 1 SD from the mean.

**Figure 3:**
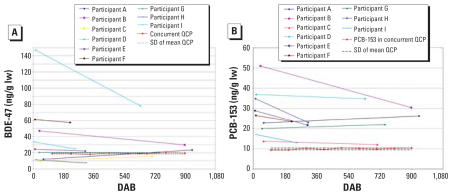
Decreases in BDE-47 (*A*) and PCB-153 (*B*) levels in breast milk from 9 LT participants collected 18–136 weeks after birth. lw, lipid weight. QCP milk samples (*n* = 8), analyzed concurrently, were plotted to illustrate measurement precision, with dotted lines showing 1 SD from the mean.

BDE-47 and PCB-153 values are plotted in Figures 2 and 3 for ST and LT mothers, respectively. Values for the eight identical QCP samples are plotted at ST collection points as if they were samples from an ST mother, with dotted lines indicating 1 SD from mean QCP. RSDs for the eight identical QCP and duplicate samples are given in Table 2. Based on analyses of identical samples (37 duplicate pairs and 8 concurrent QCP samples) for BDE-47 and PCB-153, respectively, mean duplicate RSDs are 5.3% and 3.9%, and RSDs for 8 QCP samples are 3.5% and 3.9%.

### Regression analysis of ST study

Results of fitting various statistical models are summarized in Table 3.

A simple mixed-effects model with only a random intercept and a fixed slope for the linear change in (log) BDE-47 over time since birth yields an estimated 3% decline in BDE-47 per month, with a 95% confidence interval (CI) of 2–4% decline per month (defined as 30 days; *p* < 0.001), using a robust method to calculate the variance of the estimated slope. The random effects model indicates that 99.6% of measurement variability arises from between-mother variation (i.e., within-mother correlation is 0.996). Thus, BDE-47 values differ between mothers, but repeated measures on the same mother are similar (Figure 2).

Extending the model to allow for random slopes of (log) BDE-47 against time since birth (i.e., a different slope for each mother) yields an average slope of −3% per month, essentially the same as the fixed slope result, with little variation in individual slopes (Figure 2).

Monthly declines for BDE-99 and BDE-100 were similar (2%; Table 3), and between-mother variation accounted for 99% and 99.3% of the total variation, respectively. Results using random slopes also mimic BDE-47, with slightly more variation in mothers’ slopes for BDE-99.

PCB-153 increased 0.3% per month (95% CI, −2 to 2% per month; *p* > 0.9) when only random intercepts were included, and between-mother variation accounted for 97% of total variation. With random slopes across mothers, the slopes averaged −0.6% per month, with 95% of the slopes estimated to lie between −3% and 2% per month (Table 3). The likelihood ratio test statistic for examining the evidence for random slopes is 13.7 with a nominal *p*-value of 0.001, despite the well-known conservativeness of this test (Snijders and Bosker 1999).

### Regression analysis of LT study

Statistical analyses of combined data for all LT study participants showed BDE-47 and PCB-153 levels declining, on average, 1% per month (Table 3), using random intercepts only. Between-mother variation accounted for 95% and 91% of total variation for BDE-47 and PCB-153, respectively, over these longer periods.

With random slopes, BDE-47 exhibited varying depuration slopes across mothers, with an average slope again corresponding to a decline of 1% per month (*p* = 0.035), with 95% of mothers’ slopes estimated to lie between −5% and 2% per month (only one of nine mothers had a positive slope). PCBs had less evidence for random slopes, with 95% of slopes estimated to lie between −4% and 0.8% per month, again averaging −1% per month.

### Depuration rates earlier in lactation (0–4 weeks after birth)

Using two groups of mothers from the CS CA study, we also assessed depuration rates for the 28-day period before initial breast milk samples were collected by ST mothers. One group had collected their initial samples early in lactation, 3–28 days after birth (DAB; *n* = 35), and the other group later, 29–56 DAB (*n* = 35). PBDE and PCB levels in the two groups were roughly similar (Table 1), as were levels in earlier versus later samples collected in either of the two 28-day periods (0–28 or 29–56 DAB). In plots of chemical levels compared with DAB, we found no evidence that samples collected earlier in either of the two 28-day periods (0–28 or 29–56 DAB) had higher PBDE or PCB levels. We examined and ruled out confounding by biases in collection date and region. Thus, we found no compelling evidence that depuration rates early in lactation (< 28 DAB) were higher than those we measured in the ST and LT studies later in lactation. This contrasts with a 50% decrease in polychlorinated dibenzo-*p*-dioxin (PCDD) levels in one mother between 7 and 35 DAB (Furst et al. 1989) and a “main decrease in the first [6] weeks” in 15 mothers (Beck et al. 1994).

## Discussion

### Power of the depuration study: analytical precision and sample size

The low RSD and tight SD lines for the 8 QCP samples (Figures 2–3) and the low RSDs for the 16 and 21 duplicates indicate good precision in PBDE and PCB measurements, The point-to-point variations seen in depuration profiles at the different ST sampling points have biological origins, as the variations are much larger than those seen with the 8 QCP samples (Figure 2).

Sample size is limited in both ST and LT studies. However, the precision and regularity of the data, and the collection of serial samples from each ST mother, argue that the results give accurate estimates of monthly percentage declines. The repeated measures on all 18 mothers provide considerably more reliable estimates of per-person monthly declines than an equivalently sized cross-sectional design, especially because observed rates of decline are consistent both across mothers and a wide range of contaminant levels.

### ST study (4–24 weeks after birth)

PBDE congener levels generally decreased 2–3% per month, with BDE-47 decreasing 3% per month with homogeneous slopes. These decreases held true over a 50-fold range of initial ∑PBDE levels (21–1,330 ng/g lipid weight) in the mothers. Slopes for PCB-153 were more heterogeneous than for PBDE-47, and far exceeded the small variations seen in the plot of 8 QCP samples. The random slope estimate for PCB-153 averaged −0.6% per month (95% CI, −3% to 2%; *p* = 0.40 (Table 3). The wide 95% CIs reflect variable depuration slopes and variation in individual slopes. Similar variations in PCBs have been reported for serial samples from three separate mothers (Gonzalez et al. 1995; Skaare and Polder 1990; Yakushiji et al.1978) and in PBBs from a fourth (Brilliant et al. 1978).

The larger variations in PCBs compared with PBDEs seen in some ST participants remain unexplained, but they could be due to laboratory, chance, or biology. If biology, respective half-lives seem unlikely factors, because estimated half-lives in humans for PCBs (2–5 years) and the lower brominated PBDEs (1.6–6.5 years; Geyer et al. 2004) are similar. However, differences between PBDEs and PCBs in their sources, pathways, and patterns (continuous vs. intermittent) of human exposures may contribute. Major PCB exposures are dietary (from consumption of contaminated fish or animal fats), and these exposures can be intermittent. For PBDEs, exposures may be more continuous; a major contributor to human exposures may be inhalation of indoor dusts in homes, offices, or cars, for example (Jones-Otazo et al. 2005; Schecter et al. 2005; Sjödin et al. 2003b; Stapleton et al. 2005; Thuresson et al. 2006; Wilford et al. 2005).

Diet might explain the intraindividual variations seen in PCB depuration slopes seen here and in the serial breast milk samples from a mother in Norway (Skaare and Polder 1990) and in PBBs in a Michigan mother after the Firemaster accident (Brilliant et al. 1978). PCBs in milk could reflect fluctuations in dietary PCBs, for example, an occasional meal of PCB-laden fish. However, for this to occur, dietary fat (and PCBs) must preferentially enter breast milk either directly, bypassing body fat, or preferentially from body fat. They also must comprise a significant portion of milk fat/PCBs.

### LT study (up to 136 weeks after birth)

Results of the LT study were similar to those of the ST study, although BDE-47 slopes were less homogeneous. The smaller number of observations per mother in the LT series and the longer time periods may contribute to greater variation in slopes. Concentrations of BDE-47 and PCB-153 declined in 8 and 7 of the 9 LT participants, respectively, and averaged 1% per month. These decreases are greater than the variations seen in the QCP samples (Figure 3) and suggest biological origins.

### Other depuration studies in breast milk

In a review of the limited published data on depuration of persistent chemicals via breast milk, LaKind et al. (2001) cited studies showing decreases for dioxins/furans ranging from 2–14% per month (Beck et al. 1994; Dahl et al. 1995; Furst et al. 1989, 1992; Hori 1993; Schecter et al. 1996), and 2–8% per month for PCBs (Hori 1993; Rogan et al. 1986; Schecter et al. 1998; Yakushiji et al. 1978). The decreases were generally higher than those we found in the present study for PBDEs (1–3%) and PCBs (0.6–1%) using serial samples from 18 primiparae. Preliminary data (Sjödin et al. 2005) suggest lower depuration rates for PBDEs, resembling those reported here.

### Hand-expressed versus pumped samples

Mean RSDs for BDE-47 and PCB-153 for the HE/P samples were 3.4% and 9.3%, respectively (Table 2). PCBs were 5–10% lower in 11 of 16 pumped samples compared with hand-expressed samples, suggesting that PCBs may stick to the plastic pump. The reductions were small, however, and breast pumps seem to be acceptable collection methods for breast milk.

### Design of breast milk studies

To assess the mother’s body burden of persistent, lipophilic chemicals such as PBDEs and PCBs, current designs recommend hand-expressing samples and collecting early (2–8 weeks) in lactation, which is challenging for first-time mothers with days-old infants. Our results may help simplify future breast milk studies in several ways. First, HE/P data show that breast pumps can be used for sample collection without much loss in accuracy for PBDEs and PCBs. Second, ST data show that PBDE and PCB levels change slowly over time, so that samples collected later in lactation (3–4 months) still give reasonable estimates of the mother’s body burden during pregnancy and presumed fetal exposures. Later collections would be easier on mother and infant and would make recruitment easier. Third, our precision data indicate that a single breast milk sample, properly analyzed, can accurately assess PBDE and PCB levels.

## Conclusions

Our time-series data indicate that body burdens of PBDEs and PCBs are lowered by lactation, but only slowly, averaging 1–3% per month. This was true for mothers with a 50-fold range of initial values (e.g., ∑PBDEs of 21–1,330 ng/g lipid weight). The similarities between mothers in the serial studies (ST and LT; *n* = 18) and the cross-sectional study (CS CA: *n* = 82) in demographic, physical, and lifestyle characteristics, as well as in levels and patterns of PBDEs and PCBs, suggest that depuration rates observed here may have broader applicability.

Our data indicate that 6 months of breast-feeding decreases PBDE levels in mothers by 12–18% and PCB 153 levels by approximately 4%. These rates of decrease are not higher earlier in lactation (< 28 DAB), but they may be lower later: declines in BDE-47 and PCB-153 averaged 1% per month over longer lactation periods. Consequently, 6–12 months of breast-feeding would not greatly reduce a mother’s body burden of these chemicals. If 6 months of breast-feeding reduced the levels of PBDEs and PCBs in primiparae by only 4–18%, fetal and lactational exposures for a second child would not be markedly lower than those for the first child. Women, like men, seem to have no easy way to reduce levels of PBDE and PCB contaminants. For lactating women, “pump and dump” strategies do not much reduce body burdens. Finally, PBDEs are ubiquitous indoor contaminants (homes, offices, cars, etc.). Many of us spend > 90% of our time indoors. Effective primary prevention measures to reduce exposures to PBDEs are unlikely to come from changes in “lifestyle” but rather through decreasing the use of these chemicals in consumer products.

## Figures and Tables

**Table 1:** California breast milk studies.

Study	CS CA	ST	LT	0–28 DAB^a^	29–56 DAB*a*
No.	82	9	9	35	35
Study period	Mar 2003–Nov 2005	Oct 2004–Jun 2005	Mar 2003–Sep 2005	Apr 2003–Sep 2005	Apr 2003–Nov 2005
Duration	130 weeks	24 weeks	Variable	4 weeks	4 weeks
Design	Primiparae; infant 2–8 weeks; samples hand-expressed	CS CA subset; 5–7 serial samples 4, 6, 8, 12, 16, 20, 24 weeks; 6 weeks, concurrent HE/P	CS CA subset; 2 samples: first at 1–6 weeks; second at 18–136 weeks	CS CA subset; samples collected 0–28 DAB	CS CA subset; samples collected 29–56 DAB
Demographics					
Median age (years)	31	29	32	32	30
Race (%)					
African American	9	10	5	9	6
Latina	12	10	10	9	14
Asian	5	10	5	3	6
White	71	60	80	80	66
Education (%)					
< High school graduate	4	10	0	6	0
High school graduate	8	0	0	11	6
Some college	19	30	15	14	31
College graduate	70	60	85	69	63
Median BMI	22.7	21.9	24	23.6	22.5
Family income (%)					
< $18,000	15	10	10	11	20
$18,001–$36,000	12	70	25	17	34
$36,001–$60,000	12	0	5	20	6
≥ $60,001	43	20	50	51	29
Infant sex [% (F/M)]	60/40	56/44	56/44	63/37	54/46
Median birth length (in.)	20	20.5	20	20	20.5
Median birth weight (lb)	7.5	7.5	7.4	7.7	8
Smoking status (%)					
Current smoker	3	10	5	3	3
Ex-smoker	43	40	10	37	46
Never-smoker	54	50	90	60	51
Breast-fed as child [% (yes/no)]	72/20	70/20	70/30	71/20	63/23
California residence (%)					
> 5 years	77	70	80	97	89
< 5 years	23	30	20	3	11

Abbreviations: BMI, body-mass index [weight (kg)/height (m)^2^]; DAB, days after birth.

a Depuration rates early in lactation, (0–28 DAB) were compared with rates later in lactation (29–56 DAB) using two groups of mothers from the CS CA study, in which one group collected initial samples 3–28 DAB (*n* = 35), and a second group 29–56 DAB (*n* = 35).

**Table 2:** Precision studies for breast milk.

				RSD (%)^a^		
Study	No.	∑PBDEs (ng/g lw)	∑PCBs (ng/g lw)	BDE-47	BDE-153	PCB-153
Concurrent QCPs	8	28–31	34–48	3.5	1.3	3.9
Duplicates^a^	21, 16	18–1,100	73–311	5.3	4.7	3.9
HE/P pairs	16	14–821	79–231	3.4	3.8	9.3

lw, lipid weight.

a Duplicate measurements were made in 21 samples for PBDEs, and in 16 samples for PCBs. RSDs for duplicates are means of the RSDs calculated for each of the 21 or 16 pairs of duplicate measurements; RSDs for HE/P pairs are means of the RSDs calculated for each of the 16 HE/P pairs of samples.

**Table 3:** Changes in levels of PBDEs and PCBs in ST and LT studies.

Study	Congener	Change per month (%)^a^	95% CI^a^	*p*-Value^b^	*p*-Value^c^
ST	BDE-47	−3	−2 to −4	10^−11^	0.89
	BDE-99	−2	−0.2 to −3.5	0.03	0.11
	BDE-100	−2	−1 to −3	0.0003	1
	BDE-153	−2	−1 to −3	0.001	0.36
	PCB-153^d^	0.3	−2 to 2	0.97	NA
	PCB-153^e^	−0.6	−3 to 2	0.40	0.001
LT	BDE-47	−1	−0.1 to −2	0.03	< 0.0001
	PCB-153	−1	−0.5 to −1.6	0.0001	0.07

NA, not applicable.

a Declines per month and 95% CIs are fixed slope estimates. With a random slope model, the estimate of the mean slope is identical to this fixed slope estimate (using the reported significant figures), except for PCB-153. For PCB-153, fixed slope estimates and mean random slope estimates are given separately.

b *p*-Value for test of zero slope using fixed slope/random intercept model.

c *p*-Value for the test of homogeneity of slopes in random intercept/random slope model. These *p*-values are conservative; Snijders & Bosker (1999) suggest dividing the *p*-value by 2 to address this conservativeness.

d Fixed slope estimate.

e Random slope estimate.
